# Associations between selected immune-mediated diseases and tuberculosis: record-linkage studies

**DOI:** 10.1186/1741-7015-11-97

**Published:** 2013-04-04

**Authors:** Sreeram V Ramagopalan, Raph Goldacre, Andrew Skingsley, Chris Conlon, Michael J Goldacre

**Affiliations:** 1 Department of Physiology, Anatomy and Genetics and Medical Research Council Functional Genomics Unit, University of Oxford, Parks Road, Oxford, OX1 3PT, UK; 2 Blizard Institute, Queen Mary University of London, Barts and the London School of Medicine and Dentistry, 4 Newark Street, London, E1 2AT, UK; 3 Unit of Health-Care Epidemiology, Department of Public Health, University of Oxford, Old Road, Oxford, OX3 7LF, UK; 4 Imperial College Medical School, Exhibition Road, London, SW7 2AZ, UK; 5 Department of Infectious Diseases and Microbiology, Oxford University NHS Trust, John Radcliffe Hospital, Headley Way, Headington, Oxford, OX3 9DU, UK

**Keywords:** Hospital episode statistics, Immune disease, Tuberculosis

## Abstract

**Background:**

Previous studies have suggested that there may be an association between some immune-mediated diseases and risk of tuberculosis (TB).

**Methods:**

We analyzed a database of linked statistical records of hospital admissions and death certificates for the whole of England (1999 to 2011), and a similar database (the Oxford Record Linkage Study (ORLS)) for a region of southern England in an earlier period. Rate ratios for TB were determined, comparing immune-mediated disease cohorts with comparison cohorts.

**Results:**

In the all-England dataset, there were significantly elevated risks of TB after hospital admission for the following individual immune-mediated diseases: Addison's disease, ankylosing spondylitis, autoimmune hemolytic anemia, chronic active hepatitis, coeliac disease, Crohn's disease, dermatomyositis, Goodpasture's syndrome, Hashimoto's thyroiditis, idiopathic thrombocytopenia purpura (ITP), myasthenia gravis, myxedema, pemphigoid, pernicious anemia, polyarteritis nodosa, polymyositis, primary biliary cirrhosis, psoriasis, rheumatoid arthritis, scleroderma, Sjögren's syndrome, systemic lupus erythematosus (SLE), thyrotoxicosis and ulcerative colitis. Particularly high levels of risk were found for Addison’s disease (rate ratio (RR) = 11.9 (95% CI 9.5 to 14.7)), Goodpasture’s syndrome (RR = 10.8 (95% CI 4.0 to 23.5)), SLE (RR = 9.4 (95% CI 7.9 to 11.1)), polymyositis (RR = 8.0 (95% CI 4.9 to 12.2)), polyarteritis nodosa (RR = 6.7 (95% CI 3.2 to 12.4)), dermatomyositis (RR = 6.6 (95% CI 3.0 to 12.5)), scleroderma (RR = 6.1 (95% CI 4.4 to 8.2)) and autoimmune hemolytic anemia (RR = 5.1 (95% CI 3.4 to 7.4)).

**Conclusions:**

These two databases show that patients with some immune-mediated diseases have an increased risk of TB, although we cannot explicitly state the direction of risk or exclude confounding. Further study of these associations is warranted, and these findings may aid TB screening, control and treatment policies.

## Background

About one-third of the world’s population is infected with *Mycobacterium tuberculosis*, but active tuberculosis (TB) occurs in only 5% to 10% of exposed individuals [1]. The disease may occur in any part of the body, but most commonly it is a pulmonary infection [1]. Manifestations of infection range from only a mild infiltration to disease that can be severely destructive, the balance between host defense and bacteria determining the outcome [1].

Links between other diseases and TB have also been uncovered. The risk of people infected with HIV developing TB is more than 20 times greater than that of people not infected with HIV [1]. Diabetes is associated with about a threefold increase in tuberculosis risk [2] and alcoholism is also associated with increased TB susceptibility [1].

In 1855, Thomas Addison described autopsy findings of six patients with adrenal tuberculosis, which continues to be one of the most common causes of adrenal insufficiency in the developing world [3]. Further studies have highlighted that immune-mediated diseases such as coeliac disease [4] and rheumatoid arthritis [5] are associated with an increased TB risk and there is also a suggestion that TB may precipitate systemic lupus erythematosus (SLE) [6]. The association between TB and immune-mediated disease has largely been ascribed to immunosuppressive drugs such as corticosteroids and/or tumor necrosis factor (TNF) antagonists, however this may be oversimplified.

Table 1 highlights further references of studies linking TB with immune-mediated diseases; many are single case reports and thus a relationship between TB and these diseases cannot be established.

**Table 1:** England (1999–2011): number of people in the study with each immune-mediated disease, and percentage who were female

**Immune-mediated diseases (ICD-10 codes) and reference**	**N**	**Female (%)**
Addison’s disease (E27.1) [3]	12,506	60.0
Ankylosing spondylitis (M45) [7]	30,281	30.4
Autoimmune hemolytic anemia (D59.1) [8]	9,696	54.0
Chronic active hepatitis (K73.2)	4,761	70.0
Crohn’s disease (K50) [9]	137,432	55.9
Coeliac disease (K90.0) [4]	70,300	66.5
Dermatomyositis (M33.0 to M33.1) [10]	2,903	67.1
Polymyositis (M33.2) [10]	4,130	62.3
Goodpasture’s syndrome (M31.0) [11]	1,157	49.5
Hashimoto’s thyroiditis (E06.3)	8,571	90.6
Idiopathic thrombocytopenia purpura (D69.3) [12]	31,821	54.7
Multiple sclerosis (G35) [13]	87,930	69.1
Myasthenia gravis (G70.0) [14]	11,745	49.8
Myxedema (E03.8 to E03.9) [15]	941,746	80.6
Pemphigus (L10)	2,341	57.2
Pemphigoid (L12) [16]	13,585	56.1
Pernicious anemia (D51.0)	68,679	68.4
Polyarteritis nodosa (M30.0) [17]	2,419	44.4
Primary biliary cirrhosis (K74.3) [18]	11,617	84.6
Psoriasis (L40) [19]	121,003	48.3
Rheumatoid arthritis (M05 to M06) [5]	346,804	71.1
Scleroderma (M34) [20]	12,681	81.9
Sjögren’s syndrome (M35.0)	18,791	88.6
Systemic lupus erythematosus (M32.1 to M32.9) [6]	27,519	86.7
Thyrotoxicosis (E05)	133,971	78.6
Ulcerative colitis (K51) [21]	191,977	48.7

References indicate some previous studies linking disorder with tuberculosis (TB). ICD-10 = *International Classification of Diseases*, tenth edition.

To investigate the association between TB and immune-mediated disease further, we undertook record linkage studies to determine the risk of TB specifically in individuals with selected immune-mediated diseases using an English national linked Hospital Episode Statistics (HES) dataset and a similar regional dataset.

## Methods

### Population and data

For our principal analyses, we used a dataset of English national HES (1999 to 2011), covering the population of England of about 50 million people, combined with statistical records of mortality. The HES data are statistical records of hospital care that are compiled for every episode of day case care or hospital admission in all National Health Service (NHS) hospitals in England. The death data derive from death certificates. The dataset used in this study, in which successive records for each individual were linked together (version 13yr-V06), was constructed by the Oxford record linkage group.

The basic methods were the same for the analysis of each disease and are described for rheumatoid arthritis followed by TB. A cohort of people with a record of day case care or inpatient admission for rheumatoid arthritis was constructed for those with a diagnosis of rheumatoid arthritis in any diagnostic position on the record for hospital care, by identifying the first episode of day case care, or admission, for rheumatoid arthritis during the study period. The *International Classification of Diseases*, tenth edition (ICD-10) codes used for each immune-mediated disease are shown in Table 1. The ICD codes used for TB were A15 to A19 and B90 in the tenth revision and equivalent codes in earlier editions. A reference cohort was constructed by identifying the first admission for each individual with various other, mainly minor medical and surgical conditions (listed in the footnotes to the tables), as in previous studies of disease associations undertaken by the Oxford group [24,25]. In its design, the standard epidemiological practice was followed, when hospital controls are used, of selecting a diverse range of conditions rather than relying on a narrow range (in case the latter are themselves atypical in their risk of TB). As a check, we have studied the risk of TB in the control conditions within the reference cohort to ensure that the reference cohort did not include control conditions that have atypically high or low TB rates.

People were included in the rheumatoid arthritis or reference cohort if they did not have an admission for TB either before or at the same time as the admission for rheumatoid arthritis or the reference condition. We then searched the database for any subsequent NHS hospital care for, or death from, TB in these cohorts. We included cases of TB regardless of whether TB was recorded as the main or secondary diagnosis on the hospital record, except where stated otherwise. We considered that rates of TB in the reference cohort would approximate those in the general population of the region while allowing for migration in and out of it (data on migration of individuals were not available).

### Statistical methods

We calculated rates of TB based on person-years. We took ‘date of entry’ into each cohort as the date of first admission for rheumatoid arthritis, or reference condition, and ‘date of exit’ as the date of first record of TB, death, or the end of data collection (28 February 2011), whichever was the earliest. We first calculated rates for TB, stratified and then standardized by age (in 5-year age groups), sex, calendar year of first recorded admission, region of residence, and quintile of patients’ Index of Deprivation score. Since the 1970s the UK government has calculated local measures of deprivation in England [26]. Seven domains of deprivation are combined to produce the overall index of deprivation: income deprivation, employment deprivation, health deprivation and disability, education skills and training deprivation, barriers to housing and services, living environment deprivation, and crime [26]. Each domain represents a specific form of deprivation experienced by people and each can be measured individually using a number of indicators [26].

We used the indirect method of standardization, using the combined rheumatoid arthritis and reference cohorts as the standard population. The stratum-specific rates in the combined rheumatoid arthritis and reference cohorts were then applied to the number of people in each stratum in the rheumatoid arthritis cohort, separately, and then to those in the reference cohort, to give an observed (O) and expected (E) number of people with TB in each of the two cohorts. The ratio of the standardized rate of occurrence of TB in the rheumatoid arthritis cohort was calculated relative to that in the reference cohort using the formula (O^RA^/E^RA^)/(O^ref^/E^ref^). The confidence interval for the rate ratio of TB and χ^2^ statistics for its significance were calculated as described elsewhere [27].

### Risk of immune-mediated disease after TB

We also reversed all these procedures to study the risk of rheumatoid arthritis (and each of the other immune-mediated diseases) after TB. TB was taken as the exposure cohort, rheumatoid arthritis as the outcome, and rates of rheumatoid arthritis were calculated in the TB exposure cohort and compared with those in the reference cohort, as described above. In this analysis, we excluded people from the TB or reference cohort if they had rheumatoid arthritis before their first admission for TB or the reference condition. This, together with similar exclusions described in the section above on rheumatoid arthritis as the exposure, ensured that no individual was included in the analysis of both TB as the exposure and as the outcome (that is, no one was counted twice).

### The original Oxford Record Linkage Study (ORLS)

This is a similar dataset, unique in England in its time, which commenced in 1963. We used the dataset from 1963 to 1998 and the same methods, originally developed to study disease associations in the ORLS [2] to investigate TB risks in people with immune-mediated diseases in the ORLS.

### Sensitivity analyses

We looked at the effects on the outcomes of each condition, separately, in the reference cohort. Where a reference condition appeared to be associated with the outcome, we removed this condition from the reference cohort to see if this made a difference to the results. In the great majority of the analyses, doing this made no material difference; in the two instances where it did make a difference, albeit small, we specify in Additional file 1.

### Ethical approval

The construction and analysis of the datasets were undertaken with the approval of the Central and South Bristol Research Ethics Committee (REC, reference 04/Q2006/175).

## Results

Table 1 shows the number of people in the study who were admitted to hospital with each of the selected immune-mediated diseases; it also shows the percentage of these who were female. The number of people in the reference cohort was 9,026,361 (48.0% female).

### Immune-mediated diseases followed by TB

There were significantly elevated risks of TB after hospital admission for the following individual immune-mediated diseases: Addison's disease, ankylosing spondylitis, autoimmune hemolytic anemia, chronic active hepatitis, coeliac disease, Crohn's disease, dermatomyositis, Goodpasture's syndrome, Hashimoto's thyroiditis, idiopathic thrombocytopenia purpura, myasthenia gravis, myxedema, pemphigoid, pernicious anemia, polyarteritis nodosa, polymyositis, primary biliary cirrhosis, psoriasis, rheumatoid arthritis, scleroderma, Sjögren's syndrome, SLE, thyrotoxicosis and ulcerative colitis (Table 2).

**Table 2:** England (1999 to 2011): rate ratios of tuberculosis following selected immune-mediated diseases (IMD)

**Immune-mediated disease (O and E for subsequent TB)**	**All time intervals: rate ratio (95% CI), *P *value**	**At least 1 year after admission: rate ratio (95% CI), *P *value**	**At least 5 years after admission: rate ratio (95% CI), *P *value**
Addison’s disease (85, 7.2)	11.9 (9.5 to 14.7), <0.001	11.9 (8.4 to 16.3), <0.001	11.4 (6.9 to 17.6), <0.001
Ankylosing spondylitis (75, 18)	4.2 (3.3 to 5.3), <0.001	4.1 (2.8 to 5.8), <0.001	3.9 (2.2 to 6.2), <0.001
Autoimmune hemolytic anemia (28, 5.5)	5.1 (3.4 to 7.4), <0.001	4.9 (2.5 to 8.5), <0.001	4.0 (1.1 to 10.3), 0.013
Chronic active hepatitis (13, 2.9)	4.5 (2.4 to 7.7), <0.001	5.6 (2.3 to 11.6), <0.001	5.8 (1.9 to 13.6), <0.001
Crohn’s disease (269, 76.1)	3.7 (3.2 to 4.1), <0.001	2.8 (2.3 to 3.5), <0.001	2.1 (1.5 to 2.8), <0.001
Coeliac disease (79, 34.1)	2.3 (1.9 to 2.9), <0.001	2.4 (1.6 to 3.3), <0.001	2.3 (1.4 to 3.7), <0.001
Dermatomyositis (9, 1.4)	6.6 (3.0 to 12.5), <0.001	1.8 (0.1 to 9.9), 0.9	0.0 (0.0 to 10.3), 0.8
Polymyositis (21, 2.7)	8.0 (4.9 to 12.2), <0.001	6.4 (2.8 to 12.7), <0.001	1.6 (0.0 to 8.8), 0.9
Goodpasture’s syndrome (6, 0.6)	10.8 (4.0 to 23.5), <0.001	3.9 (0.1 to 21.7), 0.6	8.4 (0.2 to 47.0), 0.3
Hashimoto’s thyroiditis (9, 4)	2.2 (1.0 to 4.3), 0.03	1.1 (0.1 to 4.0), 0.8	2.1 (0.3 to 7.4), 0.6
ITP (61, 17.7)	3.5 (2.7 to 4.5), <0.001	2.4 (1.4 to 3.7), <0.001	2.4 (1.1 to 4.6), 0.02
Multiple sclerosis (40, 48.7)	0.8 (0.6 to 1.1), 0.2	0.7 (0.4 to 1.2), 0.2	0.7 (0.3 to 1.3), 0.4
Myasthenia gravis (24, 9.1)	2.6 (1.7 to 3.9), <0.001	2.1 (1.0 to 4.0), 0.04	0.5 (0.01 to 2.6), 0.6
Myxedema (843, 566.6)	1.6 (1.5 to 1.7), <0.001	1.5 (1.4 to 1.7), <0.001	1.2 (1.0 to 1.5), 0.04
Pemphigus (2, 1.4)	1.4 (0.2 to 5.0), 0.96	1.5 (0.0 to 8.3), 0.8	0.0 (0.0 to 11.8), 0.7
Pemphigoid (17, 8.4)	2.0 (1.2 to 3.3), <0.001	1.8 (0.7 to 3.8), 0.2	1.5 (0.2 to 5.4), 0.9
Pernicious anemia (77, 40.8)	1.9 (1.5 to 2.4), <0.001	2.1 (1.5 to 2.8), <0.001	1.2 (0.6 to 2.3), 0.7
Polyarteritis nodosa (10, 1.5)	6.7 (3.2 to 12.4), <0.001	4.5 (0.9 to 13.1), 0.03	11.4 (3.1 to 29.4), <0.001
Primary biliary cirrhosis (24, 5.8)	4.1 (2.6 to 6.1), <0.001	4.2 (2.1 to 7.6), <0.001	3.1 (0.9 to 8.1), 0.05
Psoriasis (177, 69.8)	2.6 (2.2 to 3.0), <0.001	2.4 (1.9 to 3.0), <0.001	3.1 (2.2 to 4.1), <0.001
Rheumatoid arthritis (730, 249)	3.2 (3.0 to 3.5), <0.001	3.0 (2.7 to 3.4), <0.001	3.0 (2.5 to 3.5), <0.001
Scleroderma (42, 6.9)	6.1 (4.4 to 8.2), <0.001	5.3 (3.0 to 8.6), <0.001	5.0 (2.9 to 11.0), <0.001
Sjögren’s syndrome (46, 10.4)	4.4 (3.2 to 5.9), <0.001	5.1 (3.2 to 7.6), <0.001	4.0 (1.8 to 7.6), <0.001
SLE (138, 15.1)	9.4 (7.9 to 11.1), <0.001	9.1 (7.0 to 11.7), <0.001	9.1 (6.3 to 12.9), <0.001
Thyrotoxicosis (150, 74.8)	2.0 (1.7 to 2.4), <0.001	2.2 (1.7 to 2.7), <0.001	1.4 (0.8 to 2.1), 0.2
Ulcerative colitis (204, 125.2)	1.7 (1.4 to 1.9), <0.001	1.5 (1.2 to 1.8), 0.001	1.5 (1.1 to 2.0), 0.003

Table shows observed and expected (O and E) numbers of cases at all time intervals, and rate ratios and 95% confidence intervals (CIs) based on comparison with the control cohort for (a) all time intervals, (b) cases of tuberculosis (TB) first admitted at least a year after admission for IMD, and (c) cases of TB first admitted at least 5 years after admission for IMD. Rate ratios were adjusted for sex, age in 5-year bands, time period in single calendar years, region of residence and deprivation score associated with patients’ area of residence, in quintiles. Conditions used in reference cohort, with Office of Population, Censuses and Surveys (OPCS) code, fourth edition, for operations and *International Classification of Diseases*, tenth edition (ICD-10) code for diagnosis (with equivalent codes used for other coding editions) were: appendectomy (OPCS4 H01 to H03), adenoidectomy (E20), tonsillectomy (F34+F36), dilation and curettage (Q10.3+Q11.4), total hip replacement (W37 to W39), total knee replacement (W40 to W42), squint (ICD-10 H49 to H51), cataract (H25), otitis externa/media (H60 to H67), varicose veins (I83), hemorrhoids (I84), deflected septum, nasal polyp (J33+J34.2), impacted tooth and other disorders of teeth (K00 to K03), inguinal hernia (K40), ingrowing nail, toenail and other diseases of nail (L60), sebaceous cyst (L72.1), bunion (M20.1), internal derangement of knee (M23), dislocations, sprains and strains (S03, S13, S23, S33, S43, S53, S63, S73, S83, S93), selected limb fractures (S42, S52, S62, S82, S92), superficial injury and contusion (S00, S10, S20, S30, S40, S50, S60, S70, S80, S90), contraceptive management (Z30).

Particularly high levels of risk were found for Addison’s disease (rate ratio (RR) = 11.9 (95% CI, 9.5 to 14.7)), Goodpasture’s syndrome (RR = 10.8 (95% CI 4.0 to 23.5)), SLE (RR = 9.4 (95% CI 7.9 to 11.1)), polymyositis (RR = 8.0 (95% CI 4.9 to 12.2)), polyarteritis nodosa (RR = 6.7 (95% CI 3.2 to 12.4)), dermatomyositis (RR = 6.6 (95% CI 3.0 to 12.5)), scleroderma (RR = 6.1 (95% CI 4.4 to 8.2)) and autoimmune hemolytic anemia (RR = 5.1 (95% CI 3.4 to 7.4)).

We studied the occurrence of a first admission for TB at least 1 year and at least 5 years after the first admission for each immune-related disease to help establish whether any elevated risk of TB was confined to the short term or was more prolonged, although this reduced the statistical power within each time period (Table 2). This analysis was largely consistent with the all-period analysis, except that there was no significant association of dermatomyositis, Goodpasture’s syndrome, Hashimoto’s thyroiditis, and pemphigoid with TB risk first recorded at least a year after admission; and there was no significant association of myasthenia gravis, pernicious anemia, polymyositis, primary biliary cirrhosis and thyrotoxicosis with TB risk first recorded least 5 years after admission.

We also investigated the risk of respiratory and non-respiratory TB separately (Table 3). As expected, the risk of non-respiratory TB as compared to respiratory TB was much greater for patients with Addison’s disease. In general, confidence intervals for the risk of respiratory and non-respiratory TB overlapped indicating no significant differences between the types of TB. However, non-overlapping intervals indicated a higher risk of non-respiratory than respiratory TB for chronic active hepatitis, Crohn’s disease, primary biliary cirrhosis and systemic lupus erythematosus; and a higher risk of respiratory than non-respiratory TB in people with coeliac disease or polyarteritis nodosa.

**Table 3:** England (1999 to 2011): rate ratios for respiratory and non-respiratory tuberculosis (TB) for people with immune-mediated diseases

**Immune-mediated disease**	**Respiratory TB rate ratio, (95% CI)**	**Non-respiratory TB rate ratio, (95% CI)**
Addison's disease	6.5 (4.4 to 9.4)	33.0 (25.1 to 42.6)
Ankylosing spondylitis	4.6 (3.4 to 6.0)	4.7 (2.8 to 7.3)
Autoimmune hemolytic anemia	7.2 (4.6 to 10.7)	4.3 (1.4 to 10.1)
Chronic active hepatitis	3.0 (1.0 to 7.0)	9.3 (3.7 to 19.3)
Crohn's disease	3.3 (2.8 to 3.9)	5.7 (4.6 to 6.9)
Coeliac disease	2.8 (2.2 to 3.7)	1.6 (0.9 to 2.7)
Dermatomyositis	9.6 (4.2 to 19.0)	5.3 (0.6 to 19.2)
Polymyositis	8.7 (4.8 to 14.7)	8.7 (3.2 to 18.9)
Goodpasture's syndrome	11.3 (3.1 to 29.0)	15.0 (1.8 to 54.4)
Hashimoto's thyroiditis	2.9 (1.2 to 6.0)	1.8 (0.2 to 6.5)
Idiopathic thrombocytopenia purpura	3.8 (2.7 to 5.1)	5.3 (3.4 to 8.0)
Multiple sclerosis	1.0 (0.6 to 1.4)	0.8 (0.4 to 1.5)
Myasthenia gravis	3.1 (1.9 to 5.0)	2.5 (0.8 to 5.9)
Myxedema	1.6 (1.5 to 1.8)	2.0 (1.7 to 2.2)
Pemphigus	1.1 (0.0 to 6.2)	6.2 (0.8 to 22.3)
Pemphigoid	2.3 (1.2 to 4.1)	3.1 (1.0 to 7.3)
Pernicious anemia	2.2 (1.6 to 2.8)	2.4 (1.4 to 3.7)
Polyarteritis nodosa	8.6 (3.7 to 16.9)	3.0 (0.1 to 16.9)
Primary biliary cirrhosis	4.0 (2.1 to 6.8)	7.9 (4.1 to 13.8)
Psoriasis	2.9 (2.4 to 3.5)	2.5 (1.8 to 3.4)
Rheumatoid arthritis	3.6 (3.2 to 3.9)	3.2 (2.7 to 3.8)
Scleroderma	6.2 (4.0 to 9.1)	3.8 (1.5 to 7.8)
Sjögren's syndrome	4.8 (3.2 to 6.9)	4.8 (2.5 to 8.2)
Systemic lupus erythematosus	8.4 (6.6 to 10.5)	14.6 (11.3 to 18.7)
Thyrotoxicosis	2.3 (1.9 to 2.8)	2.4 (1.7 to 3.2)
Ulcerative colitis	1.7 (1.4 to 2.0)	1.8 (1.4 to 2.4)

When stratifying data by sex, there were no significant differences between males and females.

When restricting analyses to those where an admission for TB was recorded as the main diagnostic reason for admission after admission for an immune-mediated disease, there were significantly elevated risks for: Addison's disease, ankylosing spondylitis, autoimmune hemolytic anemia, chronic active hepatitis, coeliac disease, Crohn's disease, Goodpasture's syndrome, Hashimoto's thyroiditis, idiopathic thrombocytopenia purpura, polyarteritis nodosa, polymyositis, psoriasis, rheumatoid arthritis, scleroderma, SLE and ulcerative colitis (Additional file 1: Table S1).

### TB followed by immune-mediated diseases

In the TB cohort there were 53,253 individuals (43.9% female) and 9,021,678 individuals in the reference cohort (48.0% female). There were significantly elevated risks in people with TB for the following immune-mediated diseases: Addison’s disease, autoimmune hemolytic anemia, chronic active hepatitis, Crohn’s disease, Goodpasture's syndrome, idiopathic thrombocytopenia purpura, polyarteritis nodosa, polymyositis, Sjögren's syndrome, SLE and thyrotoxicosis (Table 4). When looking at risk 5 years or more after first admission for TB, significantly increased risks were present for Addison’s disease, Sjögren’s syndrome and SLE (Table 4).

**Table 4:** England (1999 to 2011): rate ratios of immune-mediated disease (IMD) following tuberculosis (TB)

**Immune-mediated disease (O and E)**	**All time intervals: rate ratio (95% CI), *P *value**	**At least 1 year after admission: rate ratio (95% CI), *P *value**	**At least 5 years after admission: rate ratio (95% CI), *P *value**
Addison’s disease (74, 9.6)	8.0 (6.3 to 10.1), <0.001	10.6 (7.4 to 14.8), <0.001	4.7 (2.2 to 8.7), <0.001
Ankylosing spondylitis (30, 32.9)	0.9 (0.6 to 1.3), 0.7	1.1 (0.5 to 1.9), 0.9	0.9 (0.3 to 1.9), 0.8
Autoimmune hemolytic anemia (16, 6.3)	2.6 (1.5 to 4.2), <0.001	2.2 (0.8 to 4.9), 0.1	0.7 (0.0 to 3.6), 1
Chronic active hepatitis (14, 2.9)	4.9 (2.6 to 8.2), <0.001	2.7 (0.7 to 7.1), 0.1	3.7 (0.4 to 13.6), 0.2
Crohn’s disease (101, 75.9)^a^	1.3 (1.1 to 1.6), 0.005	1.0 (0.8 to 1.3), 1.0	0.7 (0.4 to 1.2), 0.3
Coeliac disease (45, 45.0)	1.00 (0.7 to 1.3), 0.9	1.0 (0.6 to 1.5), 1.0	0.7 (0.3 to 1.4), 0.4
Dermatomyositis (3, 1.6)	1.9 (0.4 to 5.5), 0.5	4.4 (0.9 to 13.1), 0.03	0.0 (0.0 to 9.4), 0.9
Polymyositis (10, 3.3)	3.1 (1.5 to 5.7), 0.001	2.3 (0.5 to 6.7), 0.3	2.5 (0.3 to 9.0), 0.5
Goodpasture’s syndrome (4, 0.8)	5.1 (1.4 to 13.1), 0.003	0.0 (0.0 to 10.4), 0.8	0.0 (0.0 to 17.3), 0.5
Hashimoto’s thyroiditis (7, 6.1)	1.2 (0.5 to 2.4), 0.9	1.3 (0.3 to 3.8), 0.9	0.0 (0.0 to 2.2), 0.4
ITP (49, 22.4)	2.2 (1.6 to 2.9), <0.001	2.2 (1.3 to 3.4), 0.001	1.7 (0.8 to 3.1), 0.2
Multiple sclerosis (17, 49.2)	0.3 (0.2 to 0.6), <0.001	0.7 (0.3 to 1.2), 0.2	0.5 (0.2 to 1.2), 0.2
Myasthenia gravis (10, 10.1)	1.0 (0.5 to 1.8), 0.9	1.5 (0.6 to 3.3), 0.4	0.9 (0.1 to 3.3), 0.9
Myxedema (649, 888.8)	0.7 (0.7 to 0.8), <0.001	1.1 (1.0 to 1.2), 0.1	0.9 (0.8 to 1.0), 0.2
Pemphigus (4, 2.3)	1.8 (0.5 to 4.5), 0.4	0.9 (0.2 to 2.3), 1	0.0 (0.0 to 6.1), 0.9
Pemphigoid (12, 10.9)	1.1 (0.6 to 1.9), 0.9	1.2 (0.0 to 6.7), 0.7	2.1 (0.8 to 4.4), 0.1
Pernicious anemia (74, 63.2)	1.2 (0.9 to 1.5), 0.2	1.3 (0.9 to 1.9), 0.2	1.3 (0.8 to 2.0), 0.2
Polyarteritis nodosa (8, 1.8)	4.5 (1.9 to 9.0), <0.001	8.2 (3.0 to 18.2), <0.001	2.1 (0.1 to 11.8), 1.0
Primary biliary cirrhosis (13, 8.0)	1.6 (0.9 to 2.8), 0.1	1.8 (0.7 to 4.0), 0.2	1.0 (0.1 to 3.5), 0.8
Psoriasis (108, 119.9)	0.9 (0.7 to 1.1), 0.3	1.4 (1.0 to 1.8), 0.04	0.9 (0.5 to 1.3), 0.5
Rheumatoid arthritis (259, 271.8)^b^	0.9 (0.8 to 1.1), 0.5	1.1 (1.0 to 1.3), 0.1	1.0 (0.7 to 1.2), 0.8
Scleroderma (14, 8.8)	1.6 (0.9 to 2.7), 0.1	1.1 (0.3 to 2.9), 1	1.6 (0.4 to 4.2), 0.5
Sjögren’s syndrome (35, 17.8)	2.0 (1.4 to 2.8), <0.001	2.1 (1.2 to 3.6), <0.001	2.6 (1.4 to 4.6), <0.001
SLE (82, 23.2)	3.6 (2.9 to 4.5), <0.001	4.4 (3.1 to 6.0), <0.001	2.5 (1.3 to 4.3). <0.001
Thyrotoxicosis (141, 117.1)	1.2 (1.0 to 1.4), 0.03	1.5 (1.2 to 1.9), <0.001	1.1 (0.8 to 1.6), 0.5
Ulcerative colitis (106, 121.8)	0.9 (0.7 to 1.1), 0.2	0.9 (0.7 to 1.2), 0.5	0.9 (0.6 to 1.3), 0.6

The table shows observed numbers of cases of selected immune-mediated diseases in people with TB; showing observed and expected (O and E) numbers of cases at all time intervals, and rate ratios and 95% CIs based on comparison with the control cohort for (a) all time intervals , (b) cases of IMD first admitted at least a year after admission for TB, and (c) cases of IMD first admitted at least 5 years after admission for TB. ^a^For Crohn’s disease, the reference cohort was adjusted to remove appendectomy and hemorrhoids as these conditions were found to have elevated risks for Crohn’s disease. We present these ‘adjusted’ results in the Table. The unadjusted results for all time intervals were as follows: 101 observed, 90.8 expected, RR 1.1 (95% CI 0.9 to 1.4, *P* = 0.3). ^b^For rheumatoid arthritis, the reference cohort was adjusted to remove hip replacement and knee replacement as these operations are associated with rheumatoid arthritis in some patients. We present these ‘adjusted’ results in the Table. The unadjusted results for all time intervals were as follows: 259 observed, 337.2 expected, RR 0.8 (95% CI 0.7 to 0.9, *P* = 0.3).

When stratifying the data by sex, there were significant differences between males and females for myxedema and rheumatoid arthritis. Females with TB had rate ratios of 0.63 (0.57 to 0.7, *P* <0.001) for myxedema and 0.81 (0.68 to 0.95, *P* = 0.01) for rheumatoid arthritis. For males the rate ratios were 1.04 (0.9 to 1.18, *P* = 0.62) and 1.25 (1.03 to 1.51, *P* = 0.02) for myxedema and rheumatoid arthritis, respectively.

When restricting analyses to just those with an admission with a main diagnosis of TB, there were significantly elevated risks for: Addison's disease, chronic active hepatitis, idiopathic thrombocytopenia purpura, pernicious anemia, polyarteritis nodosa, polymyositis, rheumatoid arthritis, SLE and ulcerative colitis (Additional file 1: Table S2).

### Corroboration from the ORLS

In the ORLS, the number of individuals who entered the cohorts with immune-mediated diseases ranged from 79 with Goodpasture’s syndrome to 14,681 with rheumatoid arthritis. The number of individuals who entered the TB cohort was 7,143. There were significantly elevated risks of TB after hospital admission for the following individual immune-mediated diseases: Addison’s disease, ankylosing spondylitis, chronic active hepatitis, Crohn’s disease, pernicious anemia, rheumatoid arthritis and systemic lupus erythematosus (Table 5). The risk of TB was significantly elevated in people who had their first admission for TB at least 5 years after admission for Addison’s disease (RR = 33.2 (95% CI 14.2 to 66.2)), chronic active hepatitis (RR = 25.2 (95% CI 3.0 to 91.8)), Crohn’s disease (RR = 5.5 (95% CI 2.2 to 11.4)), rheumatoid arthritis (RR = 2.6 (95% CI 1.6 to 4.1)) and SLE (RR = 25.2 (95% CI 3.0 to 91.8)). The risk for Addison’s disease, dermatomyositis, Goodpasture’s syndrome, SLE, myxedema and chronic active hepatitis after admission for TB was significantly elevated. For example, the risk of first admission for Addison’s disease was significantly elevated 5 years after first admission for TB (RR = 6.7 (95% CI 2.6 to 14.5)).

**Table 5:** **Oxford Record Linkage Study (ORLS) (1963 to 1998)**^**a**^**: rate ratios for tuberculosis (TB) in people with immune-mediated disease (IMD) and for immune-mediated disease in people with tuberculosis**

**Exposure (earlier admission)**	**Outcome (later admission)**	**Observed**	**Expected**	**Rate ratio (95% CI)**	***P *value**
Addison's disease^†^	Tuberculosis	15	0.8	20.06 (11.18 to 33.27)	<0.001
Ankylosing spondylitis	Tuberculosis	6	1.6	3.87 (1.42 to 8.46)	0.002
Chronic active hepatitis^‡^	Tuberculosis	4	0.3	12.32 (3.35 to 31.66)	<0.001
Crohn's disease	Tuberculosis	18	4	4.61 (2.72 to 7.33)	<0.001
Pernicious anemia	Tuberculosis	25	11.1	2.28 (1.47 to 3.39)	<0.001
Rheumatoid arthritis	Tuberculosis	69	26.7	2.71 (2.09 to 3.46)	<0.001
SLE^b^	Tuberculosis	8	0.5	17.29 (7.44 to 34.22)	<0.001
Tuberculosis	Addison's disease^b^	13	1.9	7.6 (3.94 to 13.48)	<0.001
Tuberculosis	Chronic active hepatitis^c^	6	1.2	5.37 (1.93 to 12.07)	<0.001
Tuberculosis	Goodpasture's syndrome^b^	2	0.3	8.09 (0.91 to 34.08)	0.018
Tuberculosis	Myxedema^b^	36	23.1	1.57 (1.1 to 2.18)	0.009
Tuberculosis	SLE^b^	7	1.4	5.31 (2.1 to 11.21)	<0.001
Tuberculosis	Dermatomyositis^b^	3	0.4	8.18 (1.6 to 26.15)	0.001

The table shows observed numbers of cases of TB in people with selected immune-mediated diseases, and observed numbers of cases of selected immune-mediated diseases in people with TB; expected number of cases, rate ratios and 95% confidence intervals (CIs) based on comparison with the control cohort for statistically significant comparisons. ^a^Years 1963 to 1998 unless otherwise stated (span of years depends on whether the disease is separately identifiable in earlier editions of the *International Classification of Diseases* (ICD)). ^b^Cases identified from 1968 to 1998 (that is, from ICD-8 onwards). ^c^Cases identified from 1979 to 1998 (that is, from ICD-9 onwards.

## Discussion

Previous reports [4,5,10,21], combined with the results we present here, suggest that there is an association between some immune-mediated diseases and the risk of subsequent TB. Rates of subsequent TB were significantly high in both the all-England and the ORLS datasets for Addison’s disease, ankylosing spondylitis, chronic active hepatitis, Crohn’s disease, pernicious anemia, rheumatoid arthritis, and systemic lupus erythematosus. In addition, in the England dataset alone there were significantly high risks of TB following admission for autoimmune hemolytic anemia, coeliac disease, dermatomyositis, Goodpasture's syndrome, Hashimoto's thyroiditis, idiopathic thrombocytopenia purpura, myasthenia gravis, myxedema, pemphigoid, polyarteritis nodosa, polymyositis, primary biliary cirrhosis, psoriasis, scleroderma, Sjögren's syndrome, thyrotoxicosis and ulcerative colitis. A lack of significance in the ORLS dataset does not necessarily imply that the English data are uncorroborated findings; the ORLS dataset may merely be underpowered to detect these effects. Though small numbers of patients with the immune-mediated diseases, and with TB, may appear both in the ORLS and later in the Oxford element of HES, the ORLS and the much larger and later English national dataset are largely independent of each other.

There was also evidence of an increased risk for some immune-mediated diseases after TB, but the question of which disorder came first is difficult to disentangle. Information to determine the direction of the association between these diseases and TB would need incidence dates of development of each disease; and these are not available. We looked at the risks for TB first recorded after a year and after 5 years of first admission for each immune-mediated disease (and vice versa) in order to minimize the risk of detection bias or any misdiagnosis between immune-mediated diseases and subsequent recognition that they were, instead, manifestations of TB. We also did this to help interpretation of the causal direction of the associations. Of interest in this respect are Addison’s, Sjögren’s and SLE showing associations in both directions in the 5-year analysis. It is likely that the great majority of cases of Addison’s are caused by TB. There is also evidence to suggest that TB may be an infective trigger for immune-mediated disease, mechanistically by molecular mimicry, bystander activation or acting as an adjuvant [22,23].

The associations between other immune-mediated diseases, and their mechanisms, warrant further attention. One possible mechanism could be immune dysfunction predisposing to both immune-mediated disease and TB. Whether this dysfunction leads to an increased susceptibility to infection with TB or a greater risk of TB activation will be important to tease out. Another possible mechanism is the effect of treatment of autoimmune diseases on TB risk. Immunosuppressive drugs such as corticosteroids have long been associated with the risk of TB, and more recently, TNF antagonists have also been shown to increase risk [1]. In addition, patients who are on these therapies are more likely to be intensively investigated for infections, including TB, if complications arise during treatment. These are perhaps the most likely explanation for some of the associations observed. However, even when excluding individuals treated with TNF antagonists, studies have observed an increased risk for TB in patients with rheumatoid arthritis [5]. Vitamin D deficiency as a result of immune-mediated disease increasing TB risk has also been hypothesized [28].

Based on our data, screening for latent TB at immune-mediated disease diagnosis and regular timely screening thereafter may be beneficial. This will be especially true where we have identified particularly high levels of risk and/or where pre-existing data corroborate our findings. Although we have grouped these disorders together as immune mediated, these diseases are looked after by a diverse range of specialists (hematologists, rheumatologists, dermatologists, endocrinologists, hepatologists, respiratory physicians). Raising awareness of the importance of screening for TB in this context is more difficult but needs to be put in place.

A key strength of the dataset is its size with large numbers of fairly uncommon diseases. The risk of TB was studied within a single population, using the same methodology, which means that direct comparisons of risk between immune-mediated diseases can be made. The dataset has limitations. The data is based on prevalent cases of immune-mediated diseases and of TB (the first recorded hospital admission or episode of day case care for each person with each condition) rather than being a cohort with follow-up from the date of first diagnosis. Data are not recorded on patients who move out of the area covered by data collection or who are treated in hospitals outside the area. The dataset is limited to people who were admitted to hospital, or who received day case specialist care and thus there exists the potential for selection bias. This would not capture all people with each immune-mediated disease, although it should identify the great majority with subsequent diagnosed TB. We lack treatment data for immune-mediated diseases. There is very limited information on potential confounding factors such as detailed socioeconomic characteristics, other co-infections (for example, HIV with idiopathic thrombocytopenia purpura), ethnicity and smoking. The majority of individuals in the ORLS were born in Britain, and thus associations seen in the ORLS are in a reasonably homogeneous population in respect of ethnicity. The effect of making multiple comparisons needs to be considered. For this reason, we have given exact *P* values, as well as confidence intervals, so that the reader can judge the degree of significance for each immune-mediated disease and subsequent TB. It is possible that some of the associations that are significant at a level of *P* <0.05 may result from making multiple comparisons and the play of chance. This may particularly be so where there is no prior hypothesis to support the finding. However, many of the associations are very strong, and levels of significance high, and it is unlikely that most of these are attributable to chance alone. The ORLS data is also helpful in this regard: associations between several diseases and increased risks of TB were seen consistently in this smaller dataset suggesting that, taking the findings from both datasets together, these are not chance findings. The fact that we see the classic association between Addison’s and TB, and confirm previous findings of association between coeliac disease and rheumatoid arthritis and TB [4,5] adds further weight to the validity of our datasets and analyses.

## Conclusions

Our results should be regarded as speculative rather than definitive. They need further work, in different study designs, to confirm or refute the findings. Further studies should employ prospective longitudinal cohorts of individuals with TB and, separately, cohorts of individuals with immune-mediated disease to truly understand the mechanism behind the associations described. In countries where TB is uncommon the relative risks present translate into a small absolute risk increase. Nevertheless, these data may help to increase awareness among clinicians of a possible increased risk of TB in patients with autoimmune diseases. This could, in turn, lead to better screening, and treatment, for latent TB in this patient population.

## Abbreviations

E: Expected; HES: Hospital episode statistics; ITP: Idiopathic thrombocytopenia purpura; ICD: International classification of disease; NHS: National health service; O: Observed; ORLS: Oxford record linkage study; RR: Rate ratio; TB: Tuberculosis; TNF: Tumor necrosis factor; SLE: Systemic lupus erythematosus.

## Competing interests

The authors declare no competing interests.

## Authors’ contributions

MJG is the guarantor and designer of the study. RG led the analysis. SVR, AS and MJG contributed to the analysis and interpretation of the data. SVR wrote the first draft and all authors contributed to subsequent drafts and the final paper. All authors have read and approve the manuscript for publication.

## Pre-publication history

The pre-publication history for this paper can be accessed here:

http://www.biomedcentral.com/1741-7015/11/97/prepub

## Supplementary Material

Additional file 1: Table S1 Shows data analysis results for the rate ratios of primary diagnosis of tuberculosis following selected immune-mediated diseases (IMD) in England, 1999 to 2011. **Table S2. **Shows the data analysis results for the rate ratios of immune-mediated disease following a principal diagnosis of tuberculosis in England, 1999 to 2011. Click here for file
