# Supplementary material for: Associations between selected immune-mediated diseases and tuberculosis: record-linkage studies
Source: BMC Med. 2013 Apr 4;11:97. doi: 10.1186/1741-7015-11-97 (PMC3616814; doi:10.1186/1741-7015-11-97)
Supplement: Additional file 1: Table S1 — Shows data analysis results for the rate ratios of primary diagnosis of tuberculosis following selected immune-mediated diseases (IMD) in England, 1999 to 2011. Table S2. Shows the data analysis results for the rate ratios of immune-mediated disease following a principal diagnosis of tuberculosis in England, 1999 to 2011. [file 1741-7015-11-97-S1.docx]

Table S1. England (1999-2011): rate ratios and 95% confidence intervals (CIs) for a primary diagnosis of TB in people with selected immune-mediated diseases in England (1999-2011), compared with the control cohort.

| **Immune-mediated Disease**  **(O and E)** | **TB Rate Ratio (95% CI), P value** |
| --- | --- |
| Addison's disease (16, 1.3) | 12.4 (7.1-20.1), p<0.001 |
| Ankylosing spondylitis (7, 1.9) | 3.8 (1.5-7.7), p=0.001 |
| Autoimmune haemolytic anaemia (4, 1.1) | 3.7 (1.0-9.5), p=0.02 |
| Chronic active hepatitis (3, 0.7) | 4.4 (0.9-13.0), p=0.03 |
| Crohn's disease (100, 22.8) | 4.5 (3.7-5.5), p<0.001 |
| Coeliac disease (20, 7.9) | 2.6 (1.6-4.0), p<0.001 |
| Dermatomyositis (2, 0.4) | 5.2 (0.6-18.8), p=0.07 |
| Polymyositis (3, 0.6) | 5.3 (1.1-15.4), p=0.01 |
| Goodpasture's syndrome (4, 0.1) | 44.7 (12.2-114.6), p<0.001 |
| Hashimoto's thyroiditis (4, 0.7) | 5.6 (1.5-14.3), p<0.001 |
| Idiopathic thrombocytopenia purpura (24, 4.7) | 5.2 (3.3-7.7), p<0.001 |
| Multiple sclerosis (5, 10.7) | 0.5 (0.2-1.1), p=0.11 |
| Myasthenia gravis (5, 2.1) | 2.5 (0.8-5.7), p=0.09 |
| Myxoedema (6, 2.6) | 2.3 (0.8-5.0), p=0.08 |
| Pemphigus (1, 0.3) | 3.5 (0.1-19.3), p=0.69 |
| Pemphigoid (4, 1.7) | 2.4 (0.7-6.1), p=0.16 |
| Pernicious anaemia (3, 1.2) | 2.4 (0.5-7.1), p=0.26 |
| Polyarteritis nodosa (3, 0.2) | 12.4 (2.6-36.2), p<0.001 |
| Primary biliary cirrhosis (2, 1) | 2.1 (0.3-7.5), p=0.59 |
| Psoriasis (22, 9.5) | 2.3 (1.5-3.5), p<0.001 |
| Rheumatoid arthritis (94, 31.7) | 3.0 (2.5-3.7), p<0.001 |
| Scleroderma (6, 1.2) | 5.2 (1.9-11.3), p<0.001 |
| Sjogren's syndrome (2, 0.6) | 3.4 (0.4-12.2), p=0.24 |
| Systemic lupus erythematosus (17, 2.3) | 7.3 (4.3-11.7), p<0.001 |
| Thyrotoxicosis (11, 7.5) | 1.5 (0.7-2.6), p<0.001 |
| Ulcerative colitis (59, 42.9) | 1.4 (1.1-1.8), p=0.02 |

Table S2. England (1999-2011): rate ratios and 95% confidence intervals (CIs) for immune mediated disease in people admitted with a primary diagnosis of TB in England (1999-2011), compared with the control cohort.

| **Immune-mediated Disease**  **(O and E)** | **Rate Ratio (95% CI), P value** |
| --- | --- |
| Addison's disease (10, 1.7) | 6.0 (2.9-11.2), p<0.001 |
| Ankylosing spondylitis (4, 3) | 1.3 (0.4-3.4), p=0.78 |
| Autoimmune haemolytic anaemia (3, 1.4) | 2.1 (0.4-6.2), p=0.37 |
| Chronic active hepatitis (3, 0.6) | 5.4 (1.1-15.9), p=0.01 |
| Crohn's disease (36, 27.3) * | 1.3 (0.9-1.8), p=0.11 |
| Coeliac disease (14, 11.5) | 1.2 (0.7-2.0), p=0.56 |
| Dermatomyositis (1, 0.4) | 2.2 (0.1-12.7), p=0.94 |
| Polymyositis (3, 0.8) | 3.7 (0.8-11.1), p=0.06 |
| Goodpasture's syndrome (1, 0.1) | 6.8 (0.2-39.4), p=0.36 |
| Hashimoto's thyroiditis (0, 1.1) | 0 (0-3.4), p=0.56 |
| Idiopathic thrombocytopenia purpura (11, 5) | 2.2 (1.1-3.9), p=0.02 |
| Multiple sclerosis (6, 10.6) | 0.6 (0.2-1.2), p=0.21 |
| Myasthenia gravis (4, 2.6) | 1.5 (0.4-3.9), p=0.60 |
| Myxoedema (3, 3.1) | 1.0 (0.2-2.9), p=0.82 |
| Pemphigus (0, 0.4) | 0 (0-9.0), p=0.89 |
| Pemphigoid (2, 2.3) | 0.9 (0.1-3.2), p=0.87 |
| Pernicious anaemia (5, 1.5) | 3.5 (1.1-8.1), p=0.01 |
| Polyarteritis nodosa (3, 0.3) | 10.4 (2.1-31.2), p<0.001 |
| Primary biliary cirrhosis (4, 1.3) | 3.1 (0.8-7.9), p=0.06 |
| Psoriasis (9, 10.7) | 0.8 (0.4-1.6), p=0.71 |
| Rheumatoid arthritis (27, 24.7) ^†^ | 1.1 (0.7-1.6), p=0.72 |
| Scleroderma (1, 1.3) | 0.8 (0.02-4.2), p=0.88 |
| Sjogren's syndrome (0, 0.9) | 0 (0-4.2), p=0.68 |
| Systemic lupus erythematosus (18, 3.1) | 5.9 (3.5-9.4), p<0.001 |
| Thyrotoxicosis (17, 11.7) | 1.5 (0.9-2.3), p=0.16 |
| Ulcerative colitis (37, 53.1) | 0.7 (0.5-1.0), p=0.03 |

* For Crohn’s disease, the reference cohort was adjusted to remove appendicectomy and haemorrhoids. We present these ‘adjusted’ results in the Table. The unadjusted results were as follows: 36 observed, 34.3 expected, RR 1.1 (95% CI 0.7-1.5, p=0.84).

^†^ For rheumatoid arthritis, the reference cohort was adjusted to remove hip replacement and knee replacement. We present these ‘adjusted’ results in the Table. The unadjusted results were as follows: 27 observed, 48.8 expected, RR 0.8 (95% CI 0.7-0.9, p=0.3).
